# Identification and Characterization of a Potential Antimicrobial Peptide Isolated from Soil *Brevibacillus* sp. WUL10 and Its Activity against MRSA Pathogens

**DOI:** 10.3390/tropicalmed7060093

**Published:** 2022-06-07

**Authors:** Apichart Atipairin, Nuttapon Songnaka, Sucheewin Krobthong, Yodying Yingchutrakul, Thapanee Chinnawong, Thamonwan Wanganuttara

**Affiliations:** 1 School of Pharmacy, Walailak University, Nakhon Si Thammarat 80161, Thailand; apichart.at@mail.wu.ac.th (A.A.); nuttapon.so@wu.ac.th (N.S.); thapanee.ch@wu.ac.th (T.C.); 2 Drug and Cosmetic Excellence Center, Walailak University, Nakhon Si Thammarat 80161, Thailand; 3 Center for Neuroscience, Faculty of Science, Mahidol University, Bangkok 10400, Thailand; sucheewin82@gmail.com (S.K.); yodying.yin@nstda.or.th (Y.Y.); 4 National Omics Center, National Science and Technology Development Agency, Pathum Thani 12120, Thailand

**Keywords:** antimicrobial peptide, anti-MRSA, *Brevibacillus* sp., purification, WUL10

## Abstract

Methicillin-resistant *Staphylococcus aureus* (MRSA) is a severe threat to public health globally. The development of novel agents has encountered the repeated mechanism of drug resistance. This study aimed to investigate an anti-MRSA substance isolated from a promising soil bacterium. The result showed that an isolate (WUL10) was in the *Brevibacillus* genus. The minimum inhibitory concentration (MIC) of the purified substance was 1 µg/mL against *S. aureus* TISTR 517 and MRSA strains. This substance showed the bactericidal effect at the concentration of 1–2 µg/mL against these bacterial indicators. The activity of the substance retained more than 95% when encountering high temperatures and a wide range of pH, but it was sensitive to proteolytic enzymes and SDS. It was identified as a novel antimicrobial peptide (KVLVKYLGGLLKLAALMV-COOH) with the predicted structure of α-helix. The substance could rupture the cell wall of the tested pathogen. MIC and MBC of the synthesized peptide were 16 and 64 µg/mL, respectively. The difference in the activity between the isolated and synthetic peptides might be from the synergistic effects of other AMPs in the purified substance. This novel AMP would provide an advantage for further development of anti-MRSA substances to manage the situation of antibiotic resistance.

## 1. Introduction

Antimicrobial resistance is a major public health threat that causes the death of at least 700,000 people worldwide, and the mortality is predicted to increase to 10 million deaths each year by 2050 [1,2]. Antibiotics have been effective in the prevention and treatment of infectious diseases, but their excessive uses and misuses reduce the efficacy of infection control due to drug resistance [3,4]. Methicillin-resistant *Staphylococcus aureus* (MRSA) is a common antimicrobial-resistant pathogen that is developed by mutation of the *mec* gene encoding the altered penicillin-binding protein (PBP2a) and leading to a change of active site of target binding. Β-lactamase production in this strain also hydrolyzes β-lactam ring and inactivates antibiotics [5]. Available medicines used to treat such infections became restricted, resulting in an increased number of patient hospitalizations and treatment costs [6,7]. MRSA is a high-priority pathogen listed by World Health Organization (WHO) to encourage the research and development of new antibiotics in the post-antibiotic era [8].

Since the golden age of antibiotics, the secondary metabolites from microorganisms have been developed as new antibiotics [9,10]. Soil bacteria such as *streptomycetes*, *bacillus*, and *brevibacillus* are useful resources to produce active antimicrobial drugs, including streptomycin, vancomycin, bacitracin, daptomycin, erythromycin, and kanamycin [11]. These antibiotics are generally synthesized by employing multienzyme complexes such as polyketide synthases, non-ribosomal peptide synthases, or both combinations to produce antibiotics with diverse chemical modifications. Antimicrobial peptides (AMPs) are the host defense molecules that consist of 10–60 amino acids in length. They are formerly found in eukaryotes and functioned as an innate immune system that inhibits various pathogens (bacteria, viruses, fungi, and parasites) [12,13,14]. Subsequently, AMPs could be isolated from bacteria and possess unique structures and functions. Most AMPs have a net charge of +2 to +9 and contain more than 50% hydrophobic amino acids, resulting in the formation of an amphiphilic structure to bind target cells. Most AMP structures are disordered coils in aqueous solutions, but they form α-helical structures in the presence of phospholipid, lipopolysaccharide, trifluoroethanol, and sodium dodecyl sulfate micelles. In addition, AMPs also have α-helix or β-sheet or a combination of α-helix and β-sheet, and they form the ordered structure when interacting with bacterial surface or intracellular targets [12]. The structure transformations exert antimicrobial activity by binding to the negatively charged bacterial membrane, causing membrane permeability and cell lysis [15]. In addition, AMPs could interact with other intracellular targets such as bacterial nucleic acids, ribosomes, and enzymes, leading to inhibition of DNA replication or transcription, protein synthesis, cell wall synthesis, and cellular functions. As AMPs can inhibit the pathogenic bacteria through multiple actions on the cell membrane and intracellular targets, they are identified as a novel class of antibiotics to tackle multidrug-resistant bacteria, emerging from conventional antibiotics that function on a single target and extensively use for the long term [16].

*Brevibacillus* is a Gram-positive or Gram-variable, rod-shaped bacterium that can form endospore. It is reclassified from *Bacillus brevis* based on 16S rDNA sequence, and there are currently 23 species in this genus [17]. This bacterial strain is widely found in diverse environments, including soil, plants, and seawater [18]. *Brevibacillus* produces potent AMPs, and they have distinctive structures that can be classified based on their biosynthesis pathways. Mechanism of action of *Brevibacillus* AMPs is mainly through cell membrane damage that can be found in linear gramicidin, gramicidin S, tyrocidine, brevibacillin, and laterosporulin. Inhibitions of DNA and protein synthesis can also be targeted in some of *Brevibacillus* AMPs, such as edeine. WUL10 was a strain of *Brevibacillus* isolated from soil samples at a botanical park in the south of Thailand. Our previous study revealed that WUL10 produced the promising active substances against *S. aureus* and several strains of MRSA [19]. This study aimed to elucidate and characterize the properties of the anti-MRSA peptide. It will be useful to develop AMP for combating multidrug-resistant bacteria.

## 2. Materials and Methods

### 2.1. Microbial Strains and Culture Conditions

WUL10 was one of the potent isolates from a botanical garden soil. Briefly, the soil sample (10 g) was dispersed in sterile 0.85% NaCl, and the diluted sample (100 µL) was spread on MH agar. The plates were incubated at 30 °C until the colonies appeared before screening antimicrobial activity [19]. WUL10 was streaked on Mueller–Hinton (MH) agar (Titan Biotech Ltd., Rajasthan, India) and incubated at 30 °C for 24 h. *S. aureus* TISTR 517 was obtained from Thailand Institute of Scientific and Technological Research (TISTR), Thailand, whereas MRSA clinical isolates 142, 1096, and 2468 were from Maharaj Nakhon Si Thammarat Hospital, Nakhon Si Thammarat, Thailand. They were cultured on MH agar at 37 °C for 24 h. All strains were maintained in 40% glycerol at −80 °C.

### 2.2. Bacterial Morphology and Identification

WUL10 was grown on MH agar, and its morphological characteristics were observed by optical microscope (Carl Zeiss, Oberkochen, Germany) and scanning electron microscope (SEM) (Carl Zeiss, Oberkochen, Germany). SEM images were taken at a magnification of 50,000×by using the following parameters: accelerating voltage (5 kV), working distance (12 mm), and secondary electron detector. Identification of WUL10 was carried out by 16S rRNA sequencing using the following universal primers: 27F (AGAGTTTGATCCTGGCTCAG) and 1492R (GGTTACCTTGT TACGACTT). The sequencing data was analyzed by the NCBI BLAST, and the construction of the phylogenetic tree was obtained from MEGA X by using neighbor-joining analysis with 1000 bootstraps [20].

### 2.3. Determination of Growth Curve and Production Kinetics of Antimicrobial Substances

WUL10 colony was inoculated in 50 mL of half formula of Luria-Bertani (half LB) broth (Titan Biotech Ltd., Rajasthan, India), and incubated at 30 °C, 150 rpm overnight. The culture was adjusted the turbidity equivalent to 0.5 McFarland standard by 0.85% NaCl, and then, 2 mL of this preculture was transferred in 200 mL of half LB broth. The culture was shaken at 30 °C, 150 rpm for 7 days, and an aliquot of samples (2 mL) was collected at different time interval. One mL of sample was measured the cell growth by spectrophotometer at 625 nm (Thermo Fisher Scientific, Waltham, MA, USA). The remaining sample was centrifuged at 10,000× *g*, 4 °C for 30 min (Sigma-Aldrich Co., St. Louis, MO, USA), and the CFS was examined for the antimicrobial activity against all indicator strains by agar well diffusion assay. Three replicates of experiments were carried out, and the zone of inhibition was reported as mean ± SD.

### 2.4. Agar Well Diffusion Assay

The colonies of indicator strains were inoculated in 9 mL of sterile 0.85% NaCl, and the cell suspensions were adjusted the turbidity comparably to 0.5 McFarland standard. They were swabbed onto the MH agar, and the wells were punched onto the agar using a pipette tip. One hundred µL of CSF or purified substance of WUL10 was loaded into each well, and the plates were incubated at 37 °C for 24 h. Fresh half-LB broth was used as the negative control, whereas cefoxitin, oxacillin, and vancomycin were the positive controls. The experiments were performed in three independent experiments, and the inhibition zone was measured and reported as mean ± SD [21].

### 2.5. Purification of Antimicrobial Peptides

WUL10 was precultured in half-LB broth and incubated at 30 °C, 150 rpm overnight. The turbidity of the culture was adjusted equivalently to 0.5 McFarland standard. The preculture (1%) was transferred into 200 mL of half LB broth and incubated at 30 °C, 150 rpm for 24 h. The pooled culture (about 1700 mL) was collected and centrifuged at 10,000× *g*, 4 °C for 30 min. The CFS was precipitated by 50% ammonium sulfate, and the precipitate was dissolved in 50 mM ammonium acetate pH 5.0. It was dialyzed overnight at 4 °C against the same buffer, using a 3.5 kDa molecular weight cut-off dialysis bag (Thermo Fisher Scientific, Rockford, IL, USA). This fraction was subjected to a HiTrap SP column (GE Healthcare Bio-Sciences AB, Uppsala, Sweden) using a gradient elution between buffer A (50 mM ammonium acetate pH 5.0, 50 mM NaCl) and buffer B (50 mM ammonium acetate pH 5.0, 1 M NaCl). The flow rate was 3 mL/min, and UV detection was set at 214 nm. The active fraction was further purified by a C18 column (GL Sciences Inc., Tokyo, Japan) that was equilibrated with buffer A (0.1% trifluoroacetic acid). The substances were eluted from the column by a gradient of buffer B (0.1% trifluoroacetic acid, 90% acetonitrile) at a flow rate of 0.5 mL/min. The chromatogram was detected at 214 nm. The elution peaks were investigated the antimicrobial activity by agar well diffusion method against *S. aureus* TISTR 517. The purification balance sheet was calculated by which the protein concentration was measured by Bradford assay, and the antimicrobial activity by agar well diffusion assay was obtained by this Equation (1) [22];

(1) $$\mathrm{Antimicrobial}\;\mathrm{activity}\;\left(\frac{\mathrm{AU}}{\mathrm{mL}}\right)=\frac{2^{n}\times 1000}{v}$$

where n was the highest dilution that showed the inhibitory activity, and v was the volume of sample in μL that was added into the well.

### 2.6. Sodium Dodecyl Sulfate (SDS)-Polyacrylamide Gel Electrophoresis (PAGE) and Gel Overlay Assay

The purified fractions from HPLC were subjected to 15% SDS-PAGE to verify the protein purity and estimate the molecular weight. After electrophoresis, the gel was excised into two parts. One part of the gel was visualized by silver staining, whereas another part was fixed with the mixture of 25% ethanol and 5% glacial acetic acid for 1 h. The soft MH agar containing *S. aureus* TISTR 517 was poured into the fixed gel and then incubated at 37 °C overnight [14,23].

### 2.7. Minimum Inhibitory Concentration (MIC) and Minimum Bactericidal Concentration (MBC) of Antimicrobial Peptide

Determination of MIC by broth microdilution was followed the Clinical and Laboratory Standard Institute (CLSI) guidelines [24]. Cation-adjusted Mueller–Hinton broth (CAMHB) was used as a diluent. The purified substance (100 μL) was prepared by making the 2-fold dilution in the range of 0.125–32 μg/mL, and an aliquot (10 μL) of diluted suspension of the indicator strains (5 × 10^5^ CFU/mL) was added into each well. Indicator strains without sample were a positive control, while CAMHB alone was the negative control. The 96-well plates were incubated at 37 °C for 24 h and measured the bacterial growth. The lowest concentration that showed no visible growth of bacteria was considered as MIC. MBC was determined by further spreading 10 µL of sample in each well on MH agar and incubated at 37 °C overnight. The lowest concentration that did not observe the colony growth was considered as MBC.

### 2.8. Stability Study of Pure Antimicrobial Peptide

The purified substance (2.5 µg/mL) determined the stability against a number of stress conditions such as temperature, proteolytic enzymes, surfactants, and pH. The remaining activity of the substance was investigated by agar well diffusion assay, using *S. aureus* TISTR 517 and MRSA isolate 2468 as indicator strains. The untreated sample and half-LB broth were used as the positive and negative controls, respectively. Each experiment was performed in three independent experiments. Thermal sensitivity was determined by incubating the samples at 60, 80, and 100 °C for 1 h and 121 °C for 15 min. Stability toward a wide range of pH was analyzed by adjusting the pH of the sample solutions from 1.0 to 14.0 with 1.0 M HCl or 1.0 M NaOH. The samples were incubated at 37 °C for 1 h and then neutralized to pH 7.0 before testing the antimicrobial activity. Resistance to proteolytic enzymes (1 mg/mL proteinase K, lysozyme, trypsin, and α-chymotrypsin) and surfactants (1% SDS and Triton X-100) were examined by combining the samples with those agents. The samples were also incubated at 37 °C for 1 h, and additional heating at 80 °C for 10 min was used to inactivate enzymes before being subjected to agar well diffusion assay [25].

### 2.9. Scanning Electron Microscope (SEM)

The culture of *S. aureus* TISTR 517 and MRSA isolate 2468 were adjusted to the turbidity equivalent to 0.5 McFarland standard and treated with the pure substance (2.5 µg/mL) at 37 °C for 1, 3, and 6 h. The untreated strains were used as the negative control. Subsequently, the indicator cells were collected by centrifugation (10,000× *g*, 5 min, 4 °C) and placed onto a glass slide. The cells were fixed by incubating with 2.5% glutaraldehyde in 0.1 M phosphate buffer pH 7.2 at 4 °C for 24 h and washed with 0.1 M phosphate buffer pH 7.2. The cells were used to perform the post-fixation with 1% osmium tetroxide (OsO_4_) in distilled water for 1 h and were dehydrated with an increasing stepwise gradient of 20–100% ethanol for 15 min in each step. The fixed cells were dried using the critical point dryer (Quorum Technologies Ltd., Lewes, UK) and coated with gold (Cressington Scientific Instrument Ltd., Watford, UK). The cells were visualized under the scanning electron microscope at a magnification of 50,000×.

### 2.10. Peptide Sequencing and Antimicrobial Activity of the Synthetic Peptide

The peptide sequence of the purified substance of WUL10 was analyzed by the Q-Exactive HF LC-MS/MS Orbitrap system. Briefly, the protonated peptides were first desalted on-line on a reverse-phase C18 PepMap 100 trapping column before being resolved onto a C18 PepMap 100 capillary column with a 70 min gradient of 0.1% HCO_2_H/H_2_O (mobile phase (MP): A) and 0.1% HCO_2_H/CH_3_CN (MP: B) at a flow rate of 300 nL/min. Peptides were analyzed by applying a data-dependent Top5 acquisition mode followed by a higher-energy collisional dissociation (HCD) at collision energy of 28. Full-scan (MS) mass spectra were acquired from *m/z* 400 to 2000 with an AGC target set at 3 × 10^6^ ions and a resolution of 120,000. MS/MS scan was initiated when the ACG target reached 2 × 10^5^ ions and a resolution of 30,000. The raw LC-MS file from LC-MS/MS was imported to PeakX studio 10.0 (Bioinformatics Solutions Inc., Waterloo, CA, USA). The peptide ion intensity with +2 to +5 charge states was automated de novo peptide sequenced with default parameters with minor modifications. Briefly, mass error tolerance for MS and MS/MS were 15 ppm and 0.1 Da, respectively. HCD-fragmentation series on b- and y-ion series were used to predict the peptide sequence [26]. The confidence score was set at the high level of confidence to obtain a reliable set of high-precision peptides. The acceptable de novo peptide sequences were achieved by filtering average local confidence (ALC) to ≥50%. The acceptable peptide sequences with an average local confidence (ALC) threshold of ≥50% were submitted to the antimicrobial peptide scanner to analyze the probability of antimicrobial peptides [27]. A number of resulting peptides (18 synthetic peptides) were synthesized by Kelowna International Scientific Inc. (Taipei City, Taiwan) with purity of more than 85%, and they were prepared in a 2-fold dilution with CAMHB before examining the antimicrobial activity by broth microdilution. MIC and MBC were measured from 3 independent replicates.

### 2.11. Statistical Analysis

The results were analyzed using Student’s *t*-test, and *p* < 0.05 was regarded as statistically significant. All experiments were performed in three independent replicates, and the data were presented as mean ± SD.

## 3. Results

### 3.1. Cell Morphology and Phylogenetic Analysis

**Figure 1:**
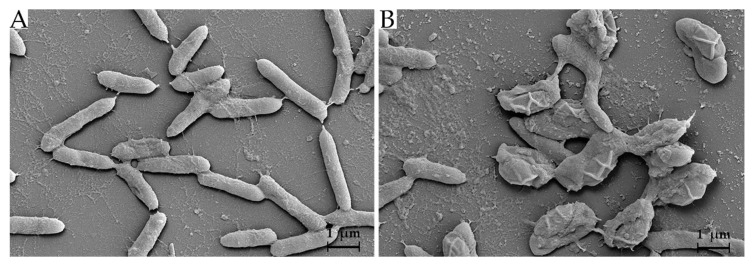
Scanning electron microgram of WUL10 isolate. (**A**) Vegetative cells and (**B**) spores were captured at 10,000× magnitude.

**Figure 2:**
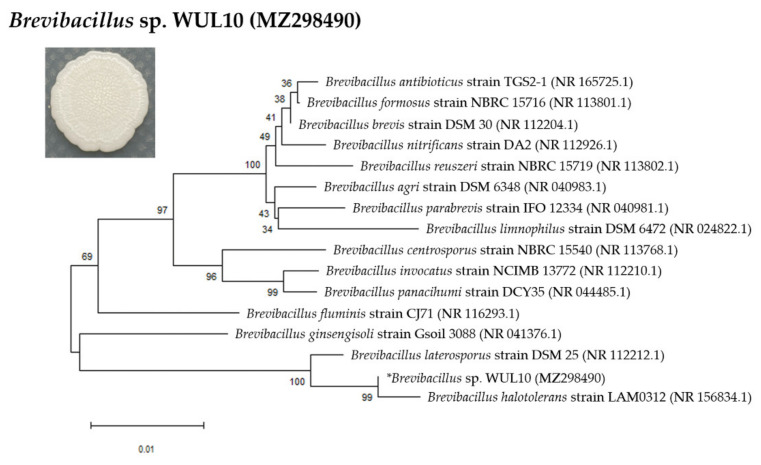
Phylogenetic tree and colony morphology of *Brevibacillus* sp. WUL10.

WUL10 appeared as a circular and white creamy colony with an undulate margin and wrinkled surface on MH agar. It was a Gram-positive bacterium. SEM images showed that its vegetative cells had rod-shaped characteristics in the width of 0.4–0.6 µm and length of 1.5–3.0 µm (Figure 1A). The spore dimension was 0.5–1.5 × 1.5–2.5 µm (Figure 1B). The vegetative cells had a smooth surface, while their spores had ellipsoidal shape with folded envelop layer. WUL10 was identified by 16S rRNA sequencing, and the partial sequence was analyzed using NCBI BLAST [28]. It belonged to the genus of *Brevibacillus* and was assigned as *Brevibacillus* sp. strain WUL10 with the accession number MZ298490. The phylogenetic tree revealed that WUL10 was closely related to *Brevibacillus halotolerans* with a sequence similarity of 99.85 (Figure 2). *Br. halotolerans* was a microorganism isolated from saline soil, and it could tolerate high salt concentrations. WUL10 and *Br. halotolerans* had similar morphology except WUL10 had no peritrichous flagella [29].

### 3.2. Production Kinetics of Antimicrobial Substances

**Figure 3:**
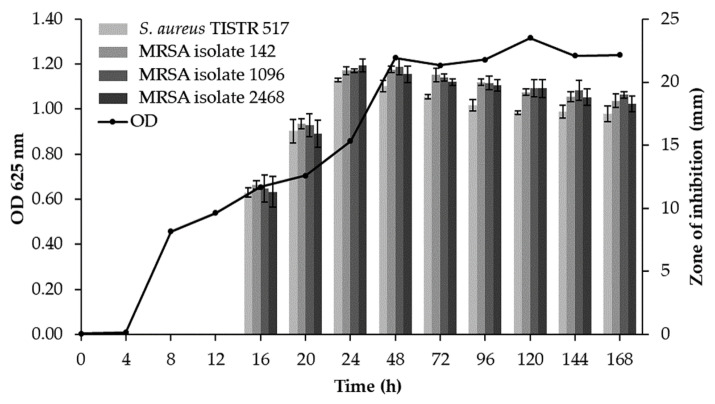
Growth curve and production kinetics of antimicrobial substances from WUL10.

The highest antimicrobial activity of CFS from WUL10 was found at 24 h of incubation, which corresponded to the late logarithmic phase of the growth curve. The inhibition zones were 20.15 ± 0.15, 20.91 ± 0.29, 20.91 ± 0.15, and 21.34 ± 0.51 mm against *S. aureus* TISTR 517 and MRSA isolate 142, 1096, and 2468, respectively. The antimicrobial activity was decreased until 168 h of cultivation, as WUL10 achieved the stationary phase (Figure 3). The potency of WUL10 supernatant at 24 h was compared to various antibiotics by the agar well-diffusion method. *S. aureus* TISTR 517 was sensitive to cefoxitin, oxacillin, and vancomycin, whereas all MRSA strains were resistant to cefoxitin and oxacillin (Table 1). Interestingly, vancomycin and WUL10 supernatant showed a broad spectrum of inhibition against *S. aureus* and its resistant strains. It indicated that WUL10 could produce the potential anti-MRSA substances, and it was beneficial to identify these bioactive agents.

### 3.3. Purification of Active Antimicrobial Substances

**Figure 4:**
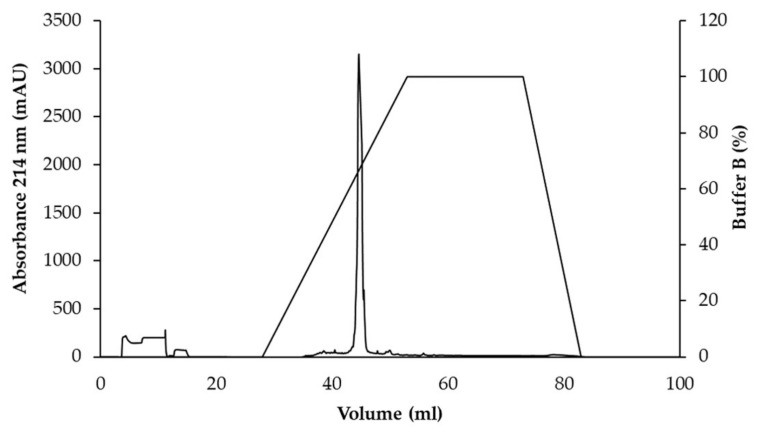
HPLC-chromatogram of an antimicrobial substance from WUL10.

**Figure 5:**
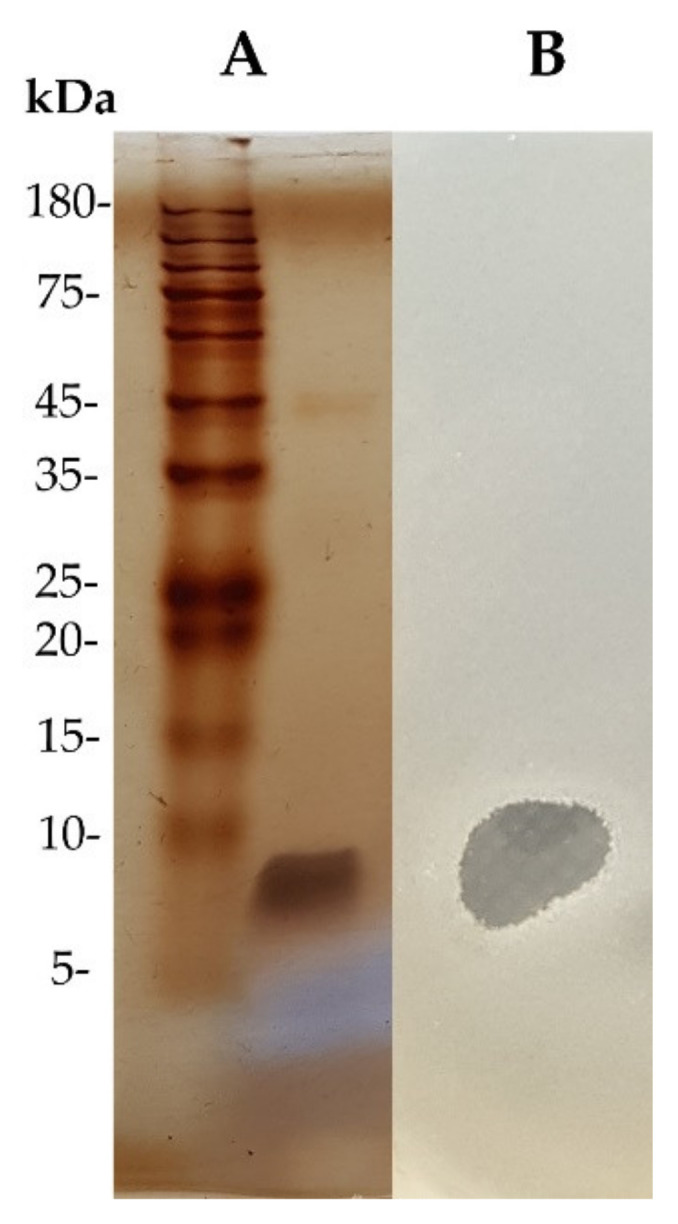
SDS-PAGE and gel overlay assay of the purified substance from WUL10. (**A**) The silver-stained gel of WUL10 substance after reversed-phase chromatography compared with protein marker. (**B**) The gel was overlaid with soft agar containing *S. aureus* TISTR 517, showing the inhibition zone at the same position.

The active substances from WUL10 were purified by ammonium sulfate precipitation, cation-exchange chromatography, and reversed-phase chromatography, respectively. The fraction in each step was tested for the antimicrobial activity against *S. aureus* TISTR 517 by the agar well-diffusion method. The result showed that the antimicrobial substances were precipitated at 50% saturation of ammonium sulfate, by which the yield remained 55.06%, and the antimicrobial activity was increased by 1.20-fold (Table 2). Then, the precipitate was purified by Hitrap SP cation exchanger, and subsequently, the active fraction was purified by an ion-pair reversed-phase column. The active substance was eluted in the main peak at about 50% acetonitrile, implying that the antimicrobial substance contained the non-polar moieties (Figure 4). At this final step, the active fractions showed a 19-fold purification with a recovery yield of 32.94%. The purified antimicrobial substance was analyzed by 15% SDS-PAGE, and the result showed a single band in the stained gel (Figure 5A). The gel was overlaid with soft agar containing *S. aureus* TISTR 517 and appeared as an inhibition zone located at the same position (Figure 5B). Taken together, it indicated that the pure substance was a potential antimicrobial peptide exhibiting anti-MRSA activity.

### 3.4. Determination of the Antimicrobial Activity of Active Substance

Purified substance from WUL10 and vancomycin had MIC of 1 and 2 µg/mL, respectively, against *S. aureus* TISTR 517 and MRSA isolate 142, 1096, and 2468, whereas cefoxitin showed MIC of 2 µg/mL against *S. aureus* TISTR 517 only. After spreading the samples in the range of MIC and supra-MIC, the WUL10 substance had MBC of 1 µg/mL on *S. aureus* TISTR 517 and MRSA isolate 2468 and that of 2 µg/mL on MRSA isolate 142 and 1096. Vancomycin showed the bactericidal activity on all indicator strains at 2 µg/mL, while cefoxitin was ineffective against MRSA (Table 3). Furthermore, several WUL10 concentrations (1×, 5×, 10×, and 20× MIC) were determined the antimicrobial activity by agar well-diffusion assay. It was found that the higher concentration of WUL10 substance exhibited a larger inhibition zone against indicator strains. The pure substance at 20X MIC (2 μg) showed the substantial activity on MRSA similar to vancomycin (30 μg), but cefoxitin (30 μg) had no activity against the resistant strains (Table 4). It indicated that WUL10 could produce an anti-MRSA peptide, and further characterizations were required to support its potential use in MRSA infection.

### 3.5. Stability Study of Purified WUL10 Substance

The stability of the purified substance of WUL10 was studied against several conditions (temperatures, pH, proteolytic enzymes, and surfactants) by measuring the remaining activity compared to the untreated samples (Table 5). The antimicrobial activity of the substance decreased when the temperature increased. However, the activity was still more than 95 percent although it was treated at 121 °C for 15 min. This indicated that the substance was thermostable. In addition, the antimicrobial activity of the substance was constant in a pH range of 1.0–11.0 against *S. aureus* TISTR 517, and it was significantly decreased at pH > 12.0 against *S. aureus* TISTR 517 and MRSA isolate 2468. Furthermore, proteinase K, trypsin, and α-chymotrypsin significantly reduced the activity, whereas lysozyme caused no effect on the antimicrobial activity of substance. SDS (1%) alone exhibited antimicrobial activity, but the combination of substance and SDS had no synergistic effect. Triton X-100 (1%) showed no activity against bacterial indicators, while the mixture of substance and Triton X-100 had unaffected activity. Taken together, the pure substance was stable at the high temperatures, but it was sensitive to proteolytic enzymes, SDS, and basic pH > 12.0. It implied that the purified substance was a small antimicrobial peptide, including aromatic and basic amino acids as the presence of the cleavage site of proteinase K, α-chymotrypsin, and trypsin.

### 3.6. Effect of Antimicrobial Substance on Bacterial Cells

**Figure 6:**
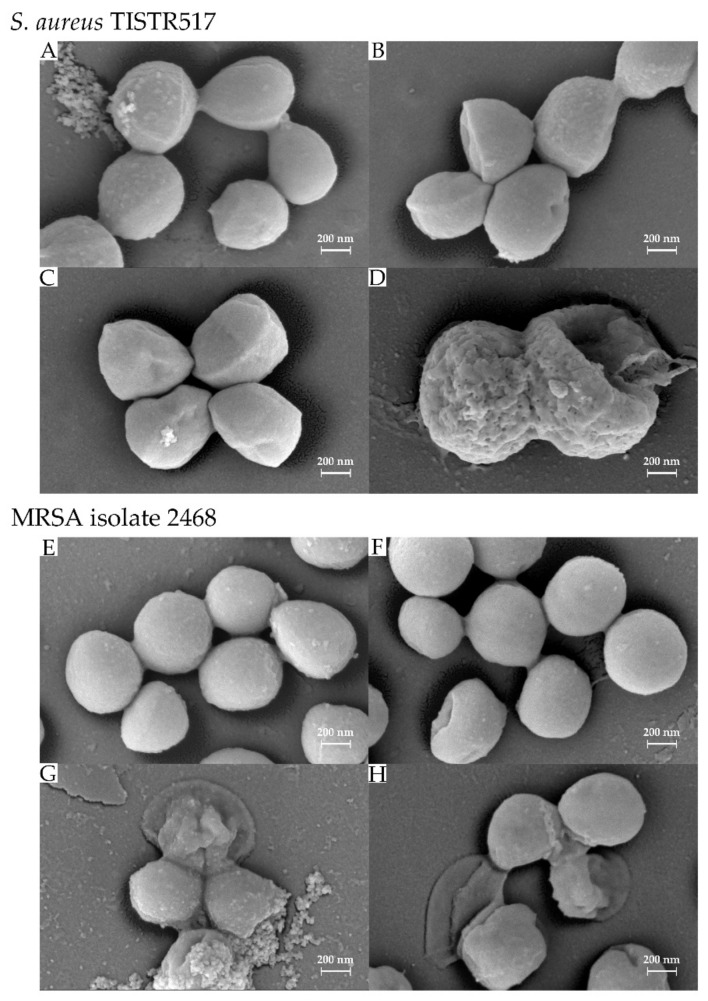
Scanning electron microscope of bacterial cells treated with the purified substance. (**A**) The untreated *S. aureus* TISTR 517 and (**B**–**D**) the effect of an antimicrobial substance on *S. aureus* TISTR 517 for 1, 3, and 6 h, respectively. (**E**) The untreated MRSA isolate 2468 and (**F**–**H**) the effect of an antimicrobial substance on MRSA isolate 2468 for 1, 3, and 6 h, respectively. The cells were captured at a magnification of 50,000×.

The SEM micrograph revealed that *S. aureus* TISTR 517 and MRSA isolate 2468 were spherical shapes with a cell dimension of 0.5–0.6 × 0.5–0.6 µm. The surface of these bacteria under the untreated conditions was firm and smooth (Figure 6A,E). However, the surface of the number of cells was collapsed and exhibited holes after treatment with the pure substance for 1 h (Figure 6B,F). The surface was rough and more collapsed when incubating with the substance for a longer time (Figure 6C,D,G,H). *S. aureus* TISTR 517 and MRSA isolate 2468 showed a leaking, rough surface after 6 and 3 h of treatment, respectively. The cells were shrunken, resulting in cell lysis. It indicated that the WUL10 substance might target the cell wall or membrane of bacteria, causing a loss of membrane integrity and then cell death.

### 3.7. Peptide Sequencing, and MIC and MBC Determination of Synthetic Peptide

**Figure 7:**
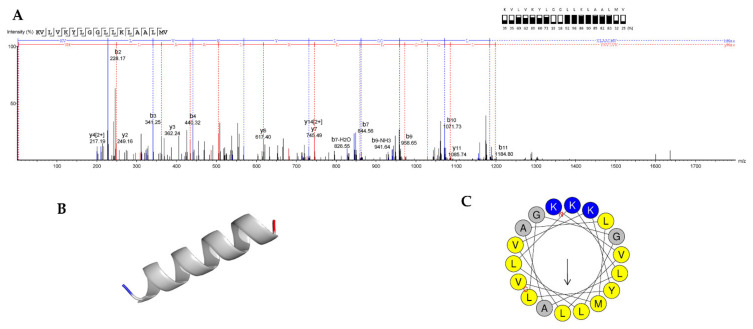
De novo peptide sequencing of the active peptide from WUL10. (**A**) fragmentation nomenclature of an antimicrobial peptide by tandem MS to generate b and y ions. (**B**) Structural prediction of KVLVKYLGGLLKLAALMV-COOH by PEP-FOLD revealed the α-helix arrangement [30,31,32]. The 3D structure was predicted by a hidden Markov model structural alphabet, having GDT-TS, Q mean, and TM values of 1.00, 0.99, and 0.89, respectively. The sOPEP energy of the structure was −39.57 kCal/mol. (**C**) Helical wheel projection of the selected peptide by HeliQuest [33]. The hydrophobic and cationic motifs are shown as yellow and blue colors, respectively. Arrow was the direction of the hydrophobic moment (μH). GDT-TS (global distance test—total score), TM (template modeling score), sOPEP (0ptimized potential for efficient protein structure prediction).

The antimicrobial peptide from WUL10 was subjected to LC-MS/MS. A de novo sequencing algorithm was used to predict the amino acid sequence originating from the parent peptide ion peak. The interesting peak of the parent peptide ion (M+2H)^2+^ was 965.1190 (*m*/*z*). The peptide sequence was fragmented by HCD fragmentation into daughter peptide ions, including the y-ion series (y_2_ = 249.16 to y_12_ = 1198.82) and b-ion series (b_2_ = 228.17 to b_11_ = 1184.80). Full HCD fragmentation series are shown in Supplementary Table S1. The peptide sequence was assigned to the MS/MS spectrum based on the difference in their mass values for a series of successive peptide b- and y-ion series, which accounted for more than 50% (Figure 7A). The sequence was identified as KVLVKYLGGLLKLAALMV-COOH, which contained seven types of amino acid residues. This antimicrobial peptide was composed of 18 residues in length with a mass of 1929.52 Da. It was predicted to have a theoretical pI of 10.00 and be cationic with a net charge of +3 at pH 7.4. Its secondary structure was predicted to be α-helix with a hydrophobicity of 0.76 and hydrophobic moment of 0.47 µH (Figure 7B,C). It was consistent with the above study that suggested the presence of aromatic and basic amino acids (tyrosine and lysine) in the sequence. The identified peptide was synthesized and determined the MIC by broth microdilution in the range of 0.5–128 µg/mL. The result showed that the MIC of the synthetic peptide was 16 µg/mL in both *S. aureus* TISTR 517 and MRSA isolate 2468. MBC of the peptide was different in that it was 16 and 64 µg/mL against sensitive and resistant strains, respectively, whereas MIC and MBC of purified natural substance from WUL10 were 1 µg/mL.

## 4. Discussion

Antimicrobial resistance is a global health crisis, as several pathogenic bacteria are becoming drug-resistant. These situations make conventional drugs less effective in treating such infections. WHO has announced a list of bacterial pathogens based on the prevalence of resistance and health severity. MRSA is categorized as a high-priority pathogen, and it encourages researchers to develop new antimicrobial agents for attenuating a crisis of multidrug-resistant bacteria [34].

Soil bacteria are valuable sources that produce several novel bioactive agents to compete with the threat of antimicrobial resistance [35]. In this study, WUL10 was identified in the *Brevibacillus* genus, and it could produce potent antimicrobial substances against *S. aureus* TISTR 517 and MRSA pathogens. Based on the phylogenetic tree, it was closely related to *Br. halotolerans* isolate LAM0312, which was isolated from saline soil in China and reported as a new species in this genus [29]. Recently, only one investigation has demonstrated that the culture of *Br. halotolerans* exhibited the larvicidal activity against *Aedes aegypti* in 48 h [36]. *Br. laterosporus* strain DSM25 was also in proximity to WUL10 and showed insecticidal, nematicidal, and antimicrobial activities [37]. Some putative genes of toxin, virulence factors, and brevibacillin synthetase were predicted in *Br. laterosporus* strains and attributed to those activities [38]. It was consistent with previous studies, showing *Brevibacillus* sp. were found in diverse environments, and they were the potential sources of many useful substances, including antimicrobial peptides such as gramicidin, laterosporulin, tostadin, daptomycin, nisin, and brevibacillin [37,39].

Presently, antimicrobial peptides are promising alternatives to conventional antibiotics for treating multidrug-resistant bacteria. They can interact with many precursor molecules, resulting in pore formation and membrane disruption. Therefore, those peptides have multiple modes of action, making them superior to antibiotics that specifically bind to one target [40]. Antimicrobial peptides can be classified based on the biosynthetic pathways involving the ribosomal or non-ribosomal syntheses [39]. Loloatins, bogorals, and brevibacillin, which were effective against MRSA strains, were produced from *Br.* *Laterosporus*. They were non-ribosomally synthesized peptides with unique characters such as linearized or cyclized molecules and conjugation of the fatty acid chain at the amino terminus [39,41]. Laterosporulin and Bac-GM100 were the ribosomally synthesized and unmodified antimicrobial peptides found in *Br. laterosporus* and *Br. brevis*, respectively.

This study demonstrated that WUL10 could produce the antimicrobial substances with the highest activity at 24 h of incubation, which corresponded to the late logarithmic phase of the growth curve. The purified substance was more tolerant towards temperature (60–121 °C) and pH (1–11) but sensitive to proteolytic enzymes, SDS and basic pH > 12.0, suggesting that the active substance from WUL10 was the stable antimicrobial peptide. It was consistent with several studies, reporting that some bacteriocin-like inhibitory substances (BLIS), such as bacillocin Bb, peanibacterin, brevican AF01, and pumilicin, were stable to heat, a wide range of pH, and proteolytic enzymes [42,43,44,45]. However, it is a challenging issue to increase the proteolytic peptide stability for clinical application. Substitution with D- or non-natural amino acids, incorporation with a methyl group at the N-terminus or cyclization on the peptide residues are utilized to protect protease enzymes [46]. Peptide conjugations with macromolecules such as antibodies, polyethylene glycol (PEG), and albumin or formulation development such as liposome and metal nanoparticles can also overcome these enzymatic problems [47].

MIC and MBC of WUL10 pure substance were 1 and 1–2 µg/mL against *S. aureus* TISTR 517 and MRSA strains, respectively. The activity was superior to vancomycin (30 μg) and cefoxitin (30 μg), as MIC and MBC of these antibiotics were 2 µg/mL, and cefoxitin was ineffective in killing MRSA because of the presence of beta-lactamase. The action of the WUL10 substance might be bactericidal because its MBC was not more than four times MIC [48]. The bactericidal activity of antimicrobial agents generally disrupts the cell membrane or interferes with vital bacterial enzymes that result in cell membrane deterioration [48]. The active substance from WUL10 was identified as KVLVKYLGGLLKLAALMV-COOH. It was a cationic peptide with a net charge of +3 at pH 7.4 and α-helix structure. The presence of lysine that possessed the cationic charge at the N-terminus coupled with the hydrophobic residues (valine, leucine, tyrosine, and alanine) enabled the insertion of this peptide with the outer surface of bacterial cells containing negatively charged lipids. These distinctive amino acid sequences facilitated the membrane disruption and exhibited the potential activity against MRSA pathogens. This antimicrobial peptide was a novel substance as it was not found in the *APD3 Antimicrobial Peptide Database* (https://aps.unmc.edu/database; 3412 peptides; accessed on 1 June 2022) and *Database of Antimicrobial Activity and Structure of Peptides* (DBAASP) (https://dbaasp.org/search; 18,994 peptides; accessed on 1 June 2022) [49,50]. Of 3412 peptides, there were 25 antimicrobial peptides obtained from bacterial sources in *APD3 Antimicrobial Peptide Database*. Multiple alignment analysis using ClustalW showed that doderlin (AP03338) was closest to WUL10 with a similarity of 16.67% (Supplementary Figure S1). Doderlin (NEPTHLLKAFSKAGFQ; 1788.01 Da) was a natural bacteriocin from *Lactobacillus acidophilus*, which was a microbial flora of gastrointestinal and urogenital tracts or found in diverse environments, and this peptide was effective against *Candida albicans* [51]. In addition, there were 18,994 peptides in the *DBAASP* database, in which 166 entries were the ribosomal peptides. Cereucin XB (MKYLGTLIKGAAGGAGAYVGEKIYNWYKN; 3135.60 Da) showed the highest similarity of 34.38% with WUL10. It was a bacteriocin from *Bacillus cereus* and exhibited low activity against *Lactococcus lactis* IL1403. The combination of cereucin XB with two peptides in the family increased the antimicrobial activity [52].

Furthermore, the MIC and MBC of this synthetic peptide against *S. aureus* TISTR 517 and MRSA isolate 2468 were 16 and 16–32 μg/mL, respectively. These concentrations were higher than those of the isolated substance (1 μg/mL). This difference in MIC and MBC between the isolated and synthetic peptides might be from the synergistic effect with other antimicrobial peptides, which were found in the purified fractionation. It was correlated with the tandem mass spectral data, showing the presence of other peptides with the length between 6 and 21 residues, molecular weight ranging from 798.53 to 2176.08 Da, and %ALC from 50 to 96. In addition, the detection limit of the LC-MS/MS instrument was also a limitation to completely identify the peptide sequences at the N- and C-terminus, which had low %ALC in each residue. A study also reviewed the key features of AMPs in the database of literature and collection of antimicrobial peptides (118 peptides) to inhibit various bacterial pathogens, including MRSA. The potential characters were short peptides (contain < 30 amino acid residues), did not contain cysteine, and possessed a net positive charge to lower the cost of chemical synthesis and target the bacterial cells, respectively [53]. Interestingly, some previous studies also investigated several synthetic AMPs against clinical MRSAs by which the peptides included the palmitoylation at the N-terminus, amidation at the C-terminus, or three or four basic amino acid residues (lysine and arginine) in the sequence. It showed that their MIC values were in the range from 1 to >128 μg/mL, and these activities were dose-dependent. However, they could not conclude that such modifications exerted the antimicrobial activity but confirmed that some examined AMPs exhibited the greater anti-MRSA activity compared to the native substances [54]. Modification of acetylation at the N-terminus of the peptide was shown to reduce the positive charge, increase the overall hydrophobic property, and improve the helix stability of the peptide. The acetylated peptide exhibited more antibacterial activity than the non-acetylated peptide, resulting from the increased peptide stability and deeper penetration to the lipid of the bacterial membrane [55].

## 5. Conclusions

In conclusion, a new antimicrobial peptide (KVLVKYLGGLLKLAALMV-COOH) was identified in *Brevibacillus* sp. WUL10. The highest activity of this substance was found after 24 h of incubation, in which it was effective against MRSA pathogens. The peptide was stable at a high temperature and a wide range of pH conditions, whereas it was sensitive to proteolytic enzymes and SDS. Structural modeling revealed that the peptide formed α-helix. The bacterial cell wall or membrane was disrupted after the treatment with this active peptide. This would provide advantageous information for further development of anti-MRSA peptides for medical applications.

## Figures and Tables

**Table 1:** Agar well diffusion of WUL10 supernatant after 24 h culture and antibiotics (means ± SD; n = 3).

Samples	Zone of Inhibition (mm ± SD)			
	*S. aureus* TISTR 517	MRSA Isolate 142	MRSA Isolate 1096	MRSA Isolate 2468
WUL10	20.15 ± 0.15	20.91 ± 0.29	20.91 ± 0.15	21.34 ± 0.51
Cefoxitin (30 µg)	32.68 ± 0.15	0.00 ± 0.00	0.00 ± 0.00	0.00 ± 0.00
Oxacillin (1 µg)	29.29 ± 0.39	0.00 ± 0.00	0.00 ± 0.00	0.00 ± 0.00
Vancomycin (30 µg)	22.94 ± 0.39	24.05 ± 0.15	23.96 ± 0.39	25.40 ± 0.25

**Table 2:** The purification balance sheet of an antimicrobial substance from WUL10.

Samples	Total Volume (mL)	Total Protein (mg)	Total Activity (AU)	Specific Activity (AU/mg)	Purification (Fold)	Yield (%)
Cell-free supernatant	1700	253.41	68,000	268.34	1.00	100.00
Ammonium sulfate precipitation	234	116.06	37,440	322.60	1.20	55.06
Cation exchange chromatography	285	10.33	22,800	2207.75	8.23	33.53
Reversed-phase chromatography	70	4.31	22,400	5201.83	19.39	32.94

**Table 3:** Microdilution assay to determine MIC and MBC of pure substance from WUL10 and standard antibiotics (n = 3).

	WUL10		Cefoxitin		Vancomycin	
Strains	MIC (µg/mL)	MBC (µg/mL)	MIC (µg/mL)	MIC (µg/mL)	MBC (µg/mL)	MIC (µg/mL)
*S. aureus* TISTR 517	1	1	2	2	2	2
MRSA isolate 142	1	2	N/D	N/D	2	2
MRSA isolate 1096	1	2	N/D	N/D	2	2
MRSA isolate 2468	1	1	N/D	N/D	2	2

N/D, not determined.

**Table 4:** Agar well-diffusion method to determine the potency of pure substance from WUL10 and antibiotics (means ± SD; n = 3).

	Zone of Inhibition (mm ± SD)			
	*S. aureus* TISTR 517	MRSA Isolate 142	MRSA Isolate 1096	MRSA Isolate 2468
Cefoxitin (30 µg)	32.77 ± 0.55	0.00 ± 0.00	0.00 ± 0.00	0.00 ± 0.00
Vancomycin (30 µg)	22.48 ± 0.23	25.61 ± 0.52	25.70 ± 0.55	26.75 ± 0.18
1× MIC WUL10 (0.1 µg)	0.00 ± 0.00	0.00 ± 0.00	9.70 ± 0.51	0.00 ± 0.00
5× MIC WUL10 (0.5 µg)	15.91 ± 0.25	17.10 ± 0.14	18.97 ± 0.39	19.43 ± 0.50
10× MIC WUL10 (1.0 µg)	19.24 ± 0.15	20.37 ± 0.26	20.73 ± 0.25	21.81 ± 0.30
20× MIC WUL10 (2.0 µg)	21.80 ± 0.15	23.03 ± 0.30	23.84 ± 0.39	25.22 ± 0.39

**Table 5:** Stability of pure substance against temperatures, proteolytic enzymes, surfactants, and pH. The result of the remaining activity when compared to the untreated sample was presented as mean ± SD from three replicate experiments.

Conditions	% Remaining Activity	
	*S. aureus* TISTR 517	MRSA Isolate 2468
Untreated sample	100.00 ± 1.21	100.00 ± 1.53
Sample at 60 °C, 1 h	98.80 ± 1.20	99.25 ± 1.77
Sample at 80 °C, 1 h	98.39 ± 1.84	99.22 ± 0.67
Sample at 100 °C, 1 h	97.58 ± 1.19 *	98.48 ± 1.32
Sample at 121 °C, 15 min	96.77 ± 0.66 *	97.72 ± 2.27
pH 1	100.81 ± 0.70	100.03 ± 2.29
pH 2	100.41 ± 0.70	101.17 ± 2.31
pH 3	100.81 ± 0.70	100.03 ± 2.29
pH 4	100.41 ± 1.40	101.17 ± 2.31
pH 5	100.41 ± 1.40	99.25 ± 1.76
pH 6	99.19 ± 0.70	98.49 ± 1.71
pH 7	100.57 ± 0.78	99.92 ± 1.26
pH 8	97.96 ± 1.40	99.23 ± 0.66
pH 9	97.96 ± 1.40	99.23 ± 0.67
pH 10	97.56 ± 2.11	98.47 ± 1.33
pH 11	97.96 ± 1.40	100.00 ± 1.16
pH 12	96.74 ± 1.40 *	98.08 ± 1.32
pH 13	96.33 ± 1.22 *	98.85 ± 0.01 *
pH 14	96.73 ± 0.72 *	98.46 ± 0.66 *
Sample + Proteinase K (1 mg/mL)	96.68 ± 0.71 *	95.77 ± 0.64 *
Sample + Lysozyme (1 mg/mL)	101.25 ± 1.25	101.16 ± 1.16
Sample + Trypsin (1 mg/mL)	97.93 ± 0.71 *	98.47 ± 0.64 *
Sample + α-chymotrypsin (1 mg/mL)	97.12 ± 1.43 *	96.59 ± 0.08 *
Sample + 1% SDS	96.30 ± 1.23 *	92.03 ± 1.25 *
Sample + 1% Triton X-100	101.65 ± 1.89	100.03 ± 2.97
Proteinase K (1 mg/mL)	0.00 ± 0.00 *	0.00 ± 0.00 *
Lysozyme (1 mg/mL)	0.00 ± 0.00 *	0.00 ± 0.00 *
Trypsin (1 mg/mL)	0.00 ± 0.00 *	0.00 ± 0.00 *
α-chymotrypsin (1 mg/mL)	0.00 ± 0.00 *	0.00 ± 0.00 *
1% SDS	95.47 ± 0.71 *	91.28 ± 1.83 *
1% Triton X-100	0.00 ± 0.00 *	0.00 ± 0.00 *

* Significance according to Student’s *t*-test, *p*-value < 0.05 compared to the untreated samples.

## Data Availability

Data are contained within the article or Supplementary Materials.

## Supplementary Materials

The following supporting information can be downloaded at: https://www.mdpi.com/article/10.3390/tropicalmed7060093/s1, Figure S1: Multiple alignment analysis of WUL10 and other antimicrobial peptides using ClustalW, Table S1: Ion table after fragmentation of antimicrobial peptide by tandem MS to generate b and y ions.
