# Supplementary material for: Identification and Characterization of a Potential Antimicrobial Peptide Isolated from Soil Brevibacillus sp. WUL10 and Its Activity against MRSA Pathogens
Source: Trop Med Infect Dis. 2022 Jun 7;7(6):93. doi: 10.3390/tropicalmed7060093 (PMC9228811; doi:10.3390/tropicalmed7060093)
Supplement: Supplementary file 1 [file tropicalmed-07-00093-s001.zip › tropicalmed-1721929-supplementary.pdf]

Supplementary Materials: Identification and Characterization of a Potential Antimicrobial Peptide Isolated from Soil *Brevibacillus* sp. WUL10 and Its Activity against MRSA Pathogens

Apichart Atipairin [1,2], Nuttapon Songnaka [1], Sucheewin Krobthong [3], Yodying Yingchutrakul [3,4] and Thapanee Chinnawong [1] and Thamonwan Wanganuttara [1,*]

(A)

(B)

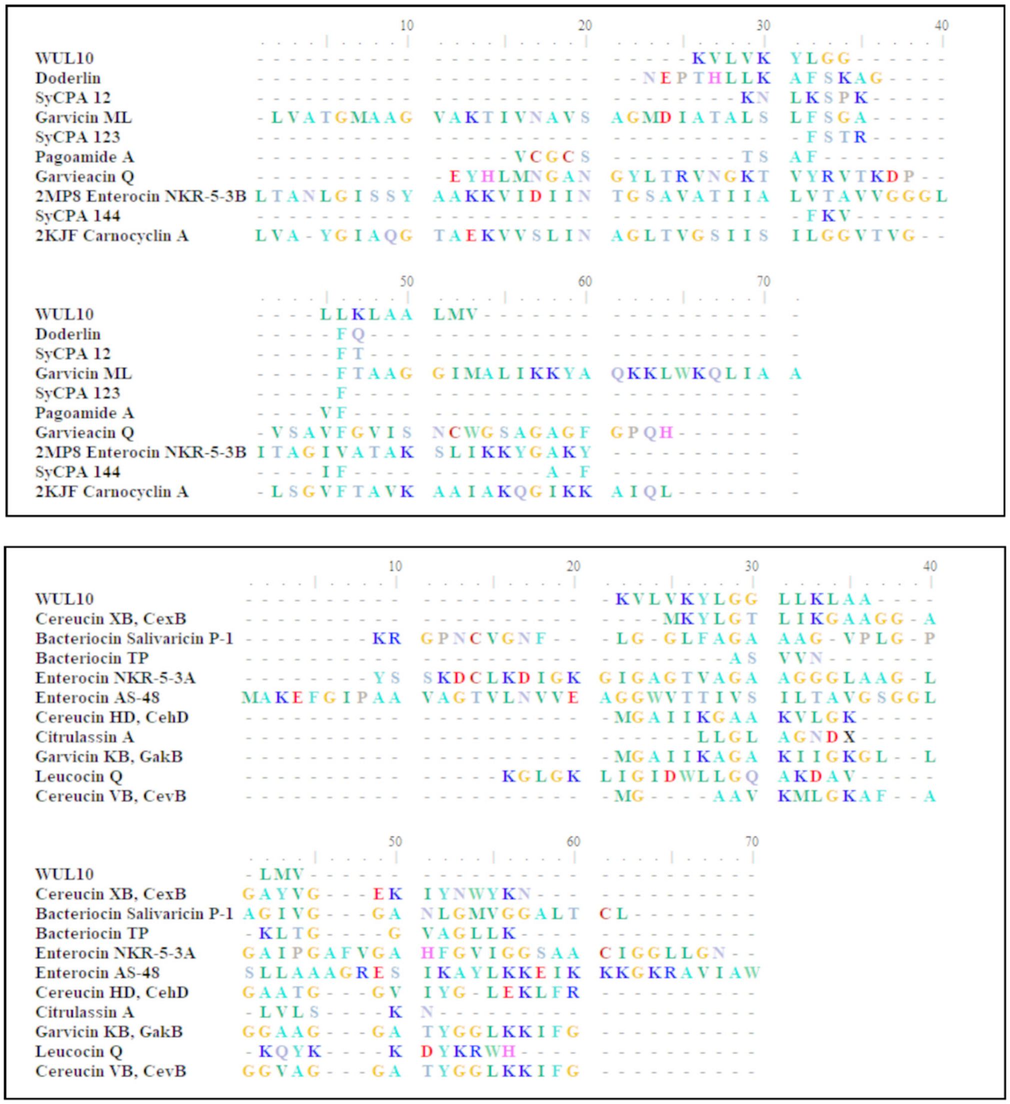

Figure S1. Multiple alignment analysis of WUL10 and other antimicrobial peptides using ClustalW. (A) APD3 Antimicrobial Peptide Database; 25 antimicrobial peptides (B) Database of Antimicrobial Activity and Structure of Peptides (DBAASP); 166 ribosomal peptides. These peptides were obtained from bacterial sources, and the first ten peptides that were similar to WUL10 were presented.

Table S1. Ion table after fragmentation of antimicrobial peptide by tandem MS to generate b and y ions.

#	b	b-H2O	b-NH3	a	c	b (2+)	Seq	y	y-H2O	y-NH3	z	z'	y (2+)	#
1	129.10	111.09	112.08	101.11	146.13	65.05	K							18
2	228.17	210.16	211.14	200.18	245.20	114.59	V	1801.14	1783.13	1784.11	1784.11	1785.12	901.07	17
3	341.25	323.24	324.23	313.26	358.28	171.13	L	1702.07	1684.06	1685.04	1685.04	1686.05	851.53	16
4	440.32	422.31	423.30	412.33	457.35	220.66	V	1588.99	1570.98	1571.96	1571.96	1572.97	794.99	15
5	568.42	550.41	551.39	540.35	585.45	284.71	K	1489.92	1471.91	1472.89	1472.89	1473.90	745.49	14
6	731.48	713.47	714.47	703.49	748.51	366.24	Y	1361.82	1343.81	1344.80	1344.80	1345.80	681.50	13
7	844.56	826.55	827.55	816.57	861.57	422.78	L	1198.82	1180.75	1181.73	1181.73	1182.74	599.88	12
8	901.59	883.58	884.56	873.59	918.61	451.29	G	1085.74	1067.66	1068.65	1068.65	1069.66	543.34	11
9	958.65	940.64	941.64	930.66	975.64	479.80	G	1028.72	1010.64	1011.63	1011.63	1012.63	514.83	10
10	1071.73	1053.71	1054.67	1043.74	1088.72	536.35	L	971.66	953.62	954.61	954.61	955.61	486.31	9
11	1184.80	1166.77	1167.75	1156.82	1201.80	592.89	L	858.58	840.54	841.52	841.52	842.53	429.77	8
12	1312.87	1294.86	1295.84	1284.88	1329.90	656.94	K	745.49	727.48	728.44	728.44	729.44	373.23	7
13	1425.96	1407.95	1408.93	1397.96	1442.98	713.47	L	617.40	599.36	600.34	600.34	601.35	309.23	6
14	1496.99	1478.98	1479.97	1469.00	1514.02	749.00	A	504.32	486.31	487.26	487.26	488.27	252.64	5
15	1568.03	1550.02	1551.00	1540.04	1585.06	784.52	A	433.24	415.24	416.22	416.22	417.23	217.19	4
16	1681.11	1663.10	1664.09	1653.12	1698.14	841.06	L	362.24	344.20	345.18	345.18	346.19	181.61	3
17	1812.15	1794.14	1795.13	1784.16	1829.18	906.58	M	249.16	231.12	232.10	232.10	233.11	125.06	2
18							V	118.09	100.08	101.06	101.06	102.07	59.54	1
